# Biosensors for Detection of Biochemical Markers Relevant to Osteoarthritis

**DOI:** 10.3390/bios11020031

**Published:** 2021-01-24

**Authors:** Umile Giuseppe Longo, Vincenzo Candela, Alessandra Berton, Sergio De Salvatore, Sara Fioravanti, Lucia Giannone, Anna Marchetti, Maria Grazia De Marinis, Vincenzo Denaro

**Affiliations:** 1 Department of Orthopedic and Trauma Surgery, Campus Bio-Medico University, Via Alvaro del Portillo, 200, Trigoria, 00128 Rome, Italy; v.candela@unicampus.it (V.C.); a.berton@unicampus.it (A.B.); s.desalvatore@unicampus.it (S.D.S.); denaro@unicampus.it (V.D.); 2 Research Unit Nursing Science, Campus Bio-Medico di Roma University, 00128 Rome, Italy; sara.fioravanti@outlook.com (S.F.); luciaagiannone@gmail.com (L.G.); a.marchetti@unicampus.it (A.M.); m.demarinis@unicampus.it (M.G.D.M.)

**Keywords:** osteoarthritis, biosensor, biomarker, ELISA, COMP, immunosensor, arthritis, cartilage, CTX-II

## Abstract

This systematic review aimed to assess the advantages of biosensors in detecting biomarkers for the early diagnosis of osteoarthritis (OA). OA is the most prevalent musculoskeletal disease and is a leading cause of disability and pain worldwide. The diagnosis of OA could be performed through clinical examinations and imaging only during the late stages of the disease. Biomarkers could be used for the diagnosis of the disease in the very early stages. Biosensors could detect biomarkers with high accuracy and low costs. This paper focuses on the biosensors mainly adopted to detect OA markers (electrochemical, optical, Quartz crystal microbalance, molecular and wearable biosensors). A comprehensive search on PubMed, Cochrane, CINAHL and Embase databases was conducted from the inception to November 2020. The Preferred Reporting Items for Systematic reviews and Meta-Analyses (PRISMA) guidelines were used to improve the reporting of the review. The Methodological Index for Non-Randomized Studies (MINORS) was used for quality assessment. From a total amount of 1086 studies identified, only 19 articles were eligible for this study. The main advantages of the biosensors reported were accuracy, limited cost and ease of use, compared to traditional methods (ELISA). Otherwise, due to the lack of data and the low level of evidence of the papers included, it was impossible to find significant results. Therefore, further high-quality studies are required.

## 1. Introduction

Osteoarthritis (OA) affects over 14 million people and constitutes a leading cause of disability and pain worldwide [1,2,3]. The incidence of OA is continuously increasing due to the ageing of the population [2,4]. The etiology of OA involves molecular, cellular and tissue modifications [1]. The progressive cartilage degeneration, subchondral sclerosis and synovial inflammation could injure other joint structures, such as ligaments and menisci [1,2]. Hands, knees, hips and spine are the most commonly injured sites [5]. Nowadays, no physical therapies or drug are proved to be effective against OA progression [6]. The diagnosis of OA is based on clinical and radiological findings (radiography, X-ray and Magnetic Resonance Imaging (MRI)) [6]. Otherwise, joint changes are only detectable in the late stages of the disease [7]. A new diagnostic method has recently been developed and consists of measuring biomarkers released by joint metabolism [8].

Biomarkers are anatomic, physiologic, biochemical or molecular parameters associated with the presence and severity of a specific condition [9]. The concentrations of OA biomarkers in body fluids could rise, reflecting the joint injury [8,10]. Consequently, low levels of biomarkers could influence the diagnosis, as they are not detectable by simple tests in mild disease [6].

Biomarker-based diagnosis is more straightforward and rapid than tissue examination or imaging diagnostic technologies [10]. Moreover, these biomarkers could also reflect the effects of medical treatments [6] and could also be detected during the early stages of OA.

Therefore, finding new methods to detect the biomarkers could improve the diagnosis and treatment of early OA [6,11]. The majority of biomarkers circulate in the synovial fluid and could be released into blood and urine [12]. Among the several available biomarkers, serum C-terminal telopeptides (sCTX), urinary C-terminal telopeptides (uCTX) and Cartilage oligomeric matrix protein (COMP) are the most accurate biomarkers for the diagnosis of OA [3,6,11]. Currently, the most common techniques adopted to assess biomarker levels are Enzyme-Linked Immunosorbent Assays (ELISA-based) [13]. Although this technique is useful, it could be performed only in a laboratory and is related to high costs [14]. The use of biosensors provides a possible solution to this limit.

For the first time, Clark and Lyons used an “enzyme electrode” biosensor in 1962 [15]. The biosensors mainly adopted to detect OA markers are electrochemical, optical, Quartz crystal microbalance (QCM), molecular and wearable biosensors [6]. Electrochemical biosensors include devices that detect biomarkers by measuring biological interactions and converting them into an electrical signal [2]. Moreover, this group includes a broad spectrum of devices depending on their method to ensure biological selectivity or their transduction method. The biosensor could be selective for a specific reaction or a macromolecule [16]. These devices are produced in different configurations (standard, nanostructured, polymer-modified and 3D printed [17]. It is possible to detect nucleic acids, enzymes, antibodies and peptides [17]. The detection methods for electrochemical biosensors include amperometry, conductometry and potentiometry [18]. Juska et al. [19] reported that electrochemical biosensors based on advanced nanostructures and miniaturized devices have high sensitivity and selectivity towards various biomarkers. The progress in nanotechnologies has led to the development of a novel sensing platform adopting nanostructures and their nanocomposites. Gold nanoparticles, carbon nanotubes, graphene quantum dots and hydrogel composites were often used as biosensors for their electrocatalytic activity [19]. The optical biosensors could detect the markers by transducing the optical signal utilizing a specific spectrum and focused wavelength. They are based on the measurement of luminescence, fluorescence and reflectance, and they could be coupled to enzyme-catalyzed reactions [20]. Nowadays, thanks to smartphones, wearable biosensors have gained attention due to their ability to track performance and parameters of individuals [21]. Wearable devices are defined as sensing devices incorporating a biological recognition element into the sensor operation (e.g., enzyme, antibody, cell receptor or organelle) [21]. Smartphone-embedded components could be used as a white light-emitting diode and illumination sensor as a light source and optical receiver [22]. QCM measures the change in frequency of a quartz resonator due to a change in mass per unit area [23]. Biomarkers as antibodies can bind terminal functional groups (-OH, -NH_2_ or -COOH) and immunocapture antigens as COMP or other targets. The QCM biosensors could detect the mass change that occurs during the binding process [5]. Molecular biosensors are devices that can measure biological processes, such as protein–protein interactions, cell and molecular trafficking or protease activity, through a signal readout [24,25].

Biosensors present several advantages compared to ELISA or other standard methods of biomarker detection. The low cost, rapid response, portability, automation and serial production possibility make biosensors useful for clinical use [26]. Recent studies have proven the utility of biosensors in rheumatoid arthritis [27,28], cancer diagnosis and pathogen detection. Lastly, for specific diseases, such as juvenile idiopathic arthritis, which require a rapid and precise diagnosis, biosensors could be the perfect solution [17].

This study aims to assess the advantages of biosensors in detecting biomarkers for the early diagnosis of OA.

## 2. Materials and Methods

**Figure 1:**
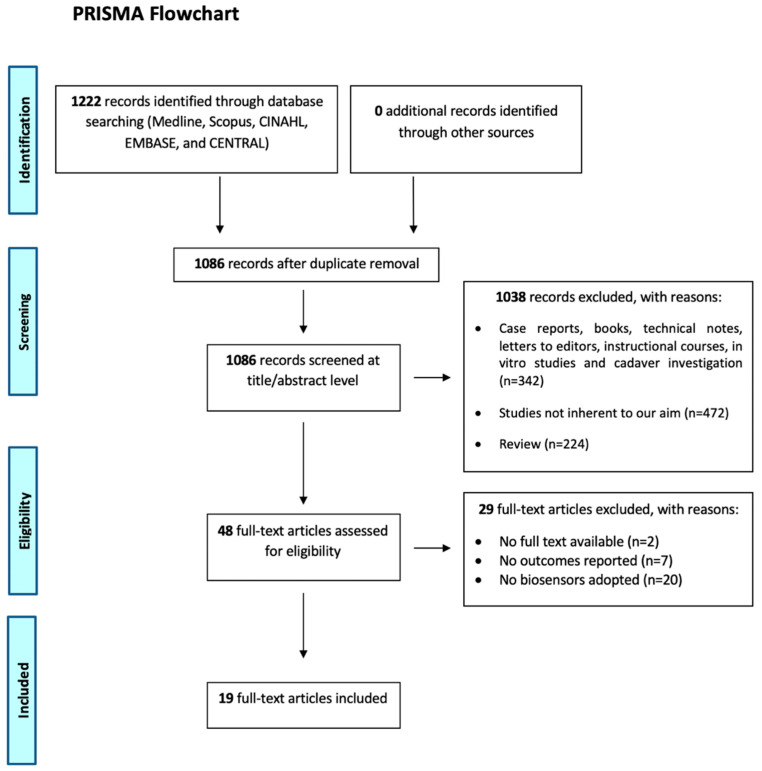
Study selection process and screening according to the Preferred Reporting Items for Systematic reviews and Meta-Analyses (PRISMA) flow chart [29].

The present paper focused on studies concerning biosensors used to assess biomarker levels for the early diagnosis of OA. The Preferred Reporting Items for Systematic reviews and Meta-Analyses (PRISMA) guidelines were used to improve the reporting of the review (Figure 1).

### 2.1. Eligibility Criteria and Search Strategy

The research question was formulated using a PICOS approach: Patient (P); Intervention (I); Comparison (C); Outcome (O) and Study design (S). This study aimed to select those articles that described patients with OA (P). The diagnosis was made by biomarkers assessed with biosensors (I), compared with those assessed with ELISA or other methods. The aim was to find biosensor advantages in terms of accuracy, rapidity of diagnosis, cost and ease of use (O). For this purpose (S), only randomized studies (RCT) and non-randomized controlled studies (NRCT), such as prospective (PS), retrospective (RS), cross-sectional (CS), observational studies (OS), case-series (CS) and case-control (CC) studies, were included.

A comprehensive search on the databases PubMed, Medline, Cochrane, CINAHL and Embase databases was conducted from the inception to November 2020 with the English language constraint. The following keywords were used isolated and combined: osteoarthritis; biomarker; biosensors; Fiber optic-particle plasmon resonance biosensor (FO-PPR); Plasmon resonance biosensor (SPRi); Fluoro-microbead guiding chip (FMGC); Matrix metalloproteinases (MIP); New fiber Bragg grating (FBG); anti-COMP; matriptase sensitive protein biosensor based on dimerization-dependent red fluorescent protein (DdRFP); fluid control device (FCD); Immunoassay with the specific antibody for uCTX-II (IDE); QCM; Biosensor based on label-free immuno-sensing with self-assembled monolayer (SAM); smartphone-embedded; illuminance; accuracy; cost; sensitivity and specificity. All the keywords were searched isolated and combined with their MeSH terms. More studies were searched among the reference lists of the selected papers. The exclusion criteria included: reviews, books and protocol studies, case reports, technical notes, letters to editors, instructional courses, in vitro and cadaver studies.

### 2.2. Study Selection and Data Collection

This systematic review was carried out in November 2020. Only English and Italian publications were included. The initial search of the article was conducted by two authors (SF and LG) using the search protocol previously described. The following research order was adopted: titles were screened first, then abstracts and full papers. A paper was considered potentially relevant and its full text reviewed if, following a discussion between the two independent reviewers, it could not be excluded based on its title and abstract. The number of articles excluded or included was registered and reported in a PRISMA flowchart (Figure 1). For designing the PRISMA, the rules by Liberati et al. were followed [29].

### 2.3. Quality Assessment

The Methodological Index for Non-Randomized Studies (MINORS) was used for quality assessment [30]. This score consists of 12 items: clearly stated aim; inclusion of consecutive patients; prospective data collection; endpoints appropriate to study aim; unbiased assessment of study endpoint; follow-up period appropriate to study aim; <5% lost to follow-up; prospective calculation of study size; adequate control group; contemporary groups; baseline equivalence of groups and adequate statistical analyses. The reviewers individually evaluated all these items. The MINORS items were scored 0 if not reported, 1 when reported but inadequate and 2 when reported and adequate. The ideal global score was 20 for NRCTs. The simplicity of MINORS comprising only 12 items makes this item readily usable by both readers and researchers. The reliability of this score has already been demonstrated [30].

Two reviewers independently evaluated (SF/LG) the potential risk of bias of the studies using the MINORS.

### 2.4. Data Synthesis and Analysis

Data were extracted and synthesized through Microsoft Excel. General study characteristics extracted were: author and year, type of study, levels of evidence, sample test, biosensor, biochemical markers, characteristics of the biomarker and advantages. Continuous variable data were reported as mean values, with the range between the minimum and maximum values. Due to the heterogeneity of the study in terms of advantages reported, only qualitative characteristics were described. Considering the heterogeneity of the included studies, it was not possible to perform a meta-analysis.

## 3. Results

According to the PRISMA protocol, a flow-chart diagram showing the selection process of the studies was reported (Figure 1). A total of 1222 studies were found (no additional studies were found in the grey literature, and no unpublished studies were retrieved). A total of 1086 studies after duplicate removal were maintained. Of that, 1038 were excluded from the study through title and abstract screening because they were not in line with our objective (*n* = 472), were study design excluded (*n* = 342) or were reviews (*n* = 224). Then, 48 full-text articles were screened. Of these studies, 29 were excluded (no full-text available = 2; no outcomes reported = 7; no biosensors adopted = 20). After this process, 19 articles were eligible for this study.

### 3.1. Study Selection and Patient Characteristics

All the studies included, excluding six articles [2,4,8,12,31,32], did not report the number of patients included and the mean follow-up. Therefore, the sample size and the follow up of the patients were not reported. For the previous reason and considering the heterogeneity of the data, a meta-analysis was not performed. No RCTs eligible for the study were found. The articles selected included 19 NRCTs (11 cross-sectional and 8 case-control). Studies were published between 2003 [12] and 2020 [2,33]. Biomarkers were found in the synovial fluid [8,12,31,32,34,35] in serum and urine [2,4,5,10,14,36,37]; in blood and uCTX-II control [8,22,38] in DNA extracted; in epithelial cells and in bovine articular cartilage [24,33,39].

The most common biosensors adopted were FOPPR [31,32,35], SPRi and FMGC biosensors [8,10,12,36]. Other biosensors used were MIP, Quartz crystal microbalance biosensor, AMPK, FBG, anti-COMP, DdRFP, FCD, IDE, QCM, SAM, hand-held optical biosensing system utilizing a smartphone-embedded illumination sensor that is integrated with the immunoblotting assay method and amperometric biosensor [2,4,5,10,14,22,24,25,33,34,37,39].

The most common biomarkers were CTX-II, both uCTX-II and sCTX-II [2,10,14,22,37,38], followed by COMP, MMP-1 and MMP-3 [4,5,8,31,32,34]. Other biomarkers adopted were: CRP, GPI, TNF, ECM, Mitochondrial DNA, Interleukin-1B, Protease matriptase and Uricase enzyme layer thickness [12,24,25,33,35,36,39].

A summary of the characteristics of the included studies is reported in Table 1.

### 3.2. Quality Assessment

All studies are NRCTs. The MINORS tool was adopted to assess the quality of evidence of the included papers. Among these studies, ten studies (55%) [5,8,10,12,25,32,33,35,38,39] had a low risk of bias, and nine studies (45%) [4,5,14,22,24,31,34,36,37] had a high risk of bias. The MINORS was reported in Table 2.

### 3.3. Results of Individual Studies

#### 3.3.1. Outcome: Accuracy

Twelve studies were included (9 cross-sectional and 3 case-control) [4,5,8,12,14,22,24,32,33,34,35]. Authors of these studies reported that biosensors have high selectivity for the detection of OA biomarkers. According to MINORS, the overall quality of evidence in these studies was assessed in the range between “low” and “high”.

#### 3.3.2. Outcome: Rapidity of Diagnosis

Eight studies were included (5 cross-sectional and 3 case-control) [2,8,10,25,32,36,37,38]. Authors of these studies reported that biosensors have faster action than traditional methods for diagnosis and treatment of OA. According to MINORS, the overall quality of evidence in these studies was assessed in the range between “low” and “high”.

#### 3.3.3. Outcome: Costs

Four studies were included (2 cross-sectional and 2 case-control) [5,22,24,31]. Authors of these studies reported that biosensors are a low-cost technology. The overall quality of evidence in these studies was assessed as “high” according to MINORS.

#### 3.3.4. Outcome: Ease of Use

Two studies were included (1 cross-sectional and 1 case-control) [5,8]. Authors of these studies reported that biosensors are easy to use for the detection of OA biomarkers. According to MINORS, the overall quality of evidence in these studies was assessed in the range between “low” and “high”.

## 4. Discussion

This study aimed to perform a systematic review of the advantages of biosensors in detecting biomarkers for early OA diagnosis.

The worldwide burden of OA is progressively increasing due to the ageing of the population [6]. It was estimated that more than 14 million people in the United States are affected by knee OA [40]. The value increases if every joint with OA is considered. Most of the studies focus on the therapy of the advanced OA stages and not on the early stage of the disease. Nowadays, it is possible to diagnose OA with clinical findings and imaging (X-ray and MRI) [6]. However, few studies focus on the possibility to detect molecular changes at the early stages of OA, before the clinical and radiological manifestation of this condition [6]. The detection of OA biomarker levels could solve this problem. Biomarkers are anatomic, physiologic, biochemical or molecular parameters associated with the presence and severity of a specific disease [4,5,8,9,36]. The concentrations of OA biomarkers in body fluids increase with the joint injury [38]. Otherwise, in the early stages of the disease, the levels of biomarkers are low, and the detection through simple tests could not be effective [37]. The gold standard for the detection of biomarkers is the ELISA test [5]. Otherwise, ELISA immunoassay is generally expansive, requires laboratory equipment, long analysis time and highly qualified operators [5,37]. It is mandatory to develop new devices for the detection of OA biomarkers [9]. The biosensors commonly adopted are enzyme-based, tissue-based, immunosensors, DNA biosensors and thermal and piezoelectric biosensors [41]. These devices are useful for detecting the low concentration of biomarkers, allowing researchers and clinicians to identify the early stages of OA [41]. Moreover, biosensors could detect the effect of medical treatment and interventions. With this feature, it is possible to adopt biosensors in diagnosis and therapy monitoring [6,8]. Biomarkers for the OA diagnosis identified in this research were: COMP, uCTX, sCTX, CRP, MMP, GPI, TNF, ECM Mitochondrial DNA, Interleukin-1B, protease matriptase and uricase enzyme layer thickness. The biosensors most commonly adopted were: FOPPR, SPRi, FMGC, MIP, Quartz crystal microbalance biosensor, AMPK, FBG, anti-COMP, DdRFP, FCD, IDE, QCM, SAM, hand-held optical biosensing system utilizing a smartphone-embedded illumination sensor that is integrated with immunoblotting assay method and amperometric biosensor.

Biosensors reported advantages in terms of accuracy, cost and ease of use. QCM biosensor and SPRi showed high accuracy for the detection of OA biomarkers [5,12,36]. Moreover, these biosensors reported advantages in terms of time compared to ELISA methods [12,22]. A hand-held optical biosensing system uses an illumination sensor embedded in the smartphone biosensor to detect uCTX-II, showing high accuracy and low production costs. Song et al. [8] reported that FMGC technology could detect uCTX-II and sCTX-II 2.5 and 3.5 times faster than the conventional ELISA method [10]. Chiang and Hsu [31,35] reported that the FOPPR biosensor is a valid alternative to ELISA because it acts in less than ten minutes and reduces the possibility of experimental errors. Moreover, Yun, Hsu and Huang [31,32,37] reported the capability of biosensors for the real-time detection of molecular interaction.

Duk Han et al. [22] described another advantage of optical biosensors. Despite ELISA being performed only in the laboratory, optical biosensors are based on the optical signal’s transduction and could be used everywhere. Unfortunately, despite advances in optical biosensing technologies, the use of commercialized optical biosensors is rare. This technology is expensive due to the requirements of high-end optical systems.

To our knowledge, this is the first systematic review on the use of biosensors for the detection of OA osteoarthrosis. Other papers focus only on biomarkers of OA, detected by different methods [3,9,42,43].

The limitations of this paper were the high heterogeneity between studies and the lack of data such as sample size or mean follow up. Moreover, due to the high heterogeneity of the data, it was impossible to perform a meta-analysis. Only English and Italian articles were included, constituting a limitation in our search string. Lastly, the quality of evidence of the studies included was low; therefore, it was impossible to obtain significant conclusions.

## 5. Conclusions

OA is a widespread disease and requires an early diagnosis to prevent joint injury. However, OA clinical diagnosis is difficult, especially in the early stages of the disease, and the lack of effective treatment can probably be attributed to the late diagnosis [5,33]. The most common methods of OA diagnosis require radiographies, exposing the patients to radiations. Therefore, it is essential to develop new specific and straightforward biosensors that could detect OA biomarkers in the early stage. This review reported the latest evidence on biosensors for OA biomarker detection, finding advantages in terms of accuracy, costs and ease of use compared to other methods.

## Figures and Tables

**Table 1:** Characteristics of the studies included and advantages of biosensors.

Author and Year	Type of Study and Level of Evidence	Sample Test	Biosensor	Biochemical Markers	Characteristics	Advantages
**Afsarimanesh 2017** [14]	Case-control study, Level III	Human serum	MIP sensor	sCTx-I	between 0.1 and 2.5 ng/mL	The proposed biosensorexhibited good selectivity and quick rebinding capacity towards target molecules.
**Ahmad 2019** [34]	Cross-sectional study, Level III	Synovial fluid	Quartz crystal microbalance biosensor.	MMP-1	Between 2 to 2000 nM	Reaction time advantage
**Chen 2018** [39]	Cross-sectional study, Level III	DNA is extracted with the D-Neasy Blood & Tissue kit	Metabolic biosensor AMPK	Mitochondrial DNA		AMPK activation limits oxidative stress and improves mitochondrial DNA integrity and function in OA chondrocytes.
**Chiang 2010** [35]	Cross-sectional study, Level III	Synovial fluid	FOPPR	Interleukin-1B	0.050–10 ng/mL	High sensitivity
**Duk Han 2014** [22]	Case-control, Level III	uCTX-II controls	Ultraviolet-visible spectroscopy	CTX-II	Detection range: 1.3–10 ng/mL	This biosensor has high sensitivity, facile fabrication, and the high obtainability and cost-effectiveness of the components used to make it
**Hartmann 2020** [33]	Cross-sectional, Level III	Bovine articular cartilage	FBG-based optoelectronic micro-indenter	ECM	5, 50, 100 and 500 μg/mL	High sensitivity
**Hsu 2011** [31]	Case-control study, Level III	Synovial fluid	FO-PPR	MMP-3		A low-cost and portable biosensor
**Huang 2013** [32]	Cross-sectional study, Level III	Synovial fluid	FO-PPR	TNF and MMP-3	TNF-a: 8.2 pg/mL; MMP-3: 8.2 pg/mL	Reaction time advantage, simple usage, high sensitivity, high selectivity
**Kim 2003** [12]	Case-control study, Level III	Synovial fluid	SPRi	GPI fused with or without NusA		Increased solubility in recombinant protein production
**Lai 2012** [4]	Cross-sectional study, Level III	Human serum	monoclonal antibodies against COMP fragments	COMP	Between 10 to 100 ng/mL	A significant increase in the COMP fragments was noted in the serum of OA patients assayed by this new sensor
**Mitchell 2018** [24]	Cross-sectional study, Level III	Epithelial cells	DdRFP;	Protease matriptase	Between 0 to 750 nM	Low cost of production, high dynamic range, robust activity under physiological and non-physiological conditions, and ideal spectroscopic properties
**Park 2015** [10]	Case-control study, Level III	Human serum and urine	FMGC; FCD	uCTX-II; SCTX-II;	uCTX-II: 200–1400 ng/mmol; sCTX-II: 0.1–2.0 ng/mL	Effectively and quantitatively assessed urinary and sCTX-II simultaneously
**Park 2016** [38]	Cross-sectional study, Level III	uCTX-II epitope-controls	Hand-held optical biosensing system utilizing a smartphone-embedded illumination sensor that is integrated with immuno-blotting assay method	uCTX-II	LOD: 0.2 ng/mL	Simple to operate, thus allowing its use by untrained and non-medical profession personnel; an immediate and accurate analysis without the use of professional equipment and special software under various ambient light conditions
**Parthasarathy 2018** [25]	Cross-sectional study, Level III	Not reported	Amperometric biosensor	Uricase enzyme layer thickness		Diagnosis can be made by seeing the change Uricase enzyme layer thickness
**Song 2011** [8]	Cross-sectional study, Level III	Human blood and synovial fluid	FMGC	COMP	Between 4 and 128 ng/mL	Ease and accuracy of biomarker quantification over a clinically important concentration range. Reaction time advantage
**Vance 2014** [36]	Cross-sectional study, Level III	Human serum	Ultrasensitive SPRi biosensors	CRP	5 fg/mL	Ultra-sensitiveSPRi biosensors offer fast turnaround time and a stronger support structure for the capture probe
**Wang 2020** [2]	Case-control study, Level III	Urine	IDE	uCTX-II	Between 10 and 100 pM	uCTX-II has been found to be a rapidly potential biomarker for OA.
**Wang 2010** [5]	Cross-sectional study, Level III	Urine	QCM	COMP	Range 1–200 ng/mL	A highly sensitive, user-friendly and cost-effective analytical method for early-stage diagnosis
**Yun 2009** [37]	Case-control study, Level III	Urine	SAM	CTX-II	Between 3 μg/mL to 50 ng/mL	Reaction time advantages

OA: osteoarthritis; RA: rheumatoid arthritis; CRP: C-reactive protein; sCTx-I: Serum C-terminal telopeptide of type I collagen; MMP-1: Matrix metalloproteinases; MIP: Molecular Imprinted Polymer sensor; AMPK: AMP-activated protein kinase; MMP-3: Proteins of the matrix metalloproteinase; TNF-a: Tumor necrosis factor.; SAM: Biosensor based on label-free immuno-sensing with self-assembled monolayer; QCM: Quartz crystal microbalance; COMP: Cartilage oligomeric matrix protein; IDE: Immunoassay with the specific antibody for uCTX-II; uCTX-II: Urinary C-terminal telopeptide fragment of type II collagen; sCTX-II: Serum C-terminal telopeptide fragment of type II collagen; FMGC: Fluoro-microbead guiding chip; FCD: fluid control device; DdRFP: matriptase sensitive protein biosensor based on dimerization-dependent red fluorescent protein; SPRi: Plasmon resonance biosensor; GPI: antibodies against glucose 6-phosphate isomerase; FO-PPR: Fiber optic-particle plasmon resonance biosensor; FBG: New fiber Bragg grating; ECM: Articular cartilage extracellular matrix.

**Table 2:** MINORS score of the included studies.

Author	Clearly Stated Aim	Inclusion of Consecutive Patients	Prospective Data Collection	Endpoints Appropriate to Study Aim	Unbiased Assessment of Study Endpoint	Follow-Up Period Appropriate to Study Aim	<5% Lost to Follow-Up	Prospective Calculation of Study Size	Adequate Control Group	Contemporary Groups	Baseline Equivalence of Groups	Adequate Statistical Analyses	Total Score (…/24)
**Afsarimanesh, 2017**	2	NA	0	2	2	0	0	0	2	2	0	0	10
**Ahmad, 2019**	2	2	0	2	1	0	0	NA	2	2	0	0	11
**Chen, 2018**	2	0	0	2	2	0	0	0	2	2	0	2	12
**Chiang, 2010**	2	2	0	2	2	0	0	0	2	2	0	2	14
**Duk Han, 2014**	2	2	0	2	2	0	NA	0	2	2	0	0	12
**Hartmann, 2020**	2	2	0	2	2	0	0	0	2	2	0	2	14
**Hsu, 2011**	2	2	NA	2	2	0	0	0	2	2	0	0	12
**Huang, 2013**	2	2	0	2	0	0	0	0	2	2	0	2	12
**Kim, 2003**	2	0	2	2	2	0	0	0	2	2	0	2	14
**Lai, 2012**	2	2	0	2	2	0	0	2	2	2	NA	2	16
**Mitchel, 2018**	2	0	0	2	2	NA	NA	NA	2	2	0	2	12
**Park, 2015**	2	2	0	2	0	0	0	0	2	2	0	0	10
**Park, 2016**	2	2	0	2	0	0	0	0	2	2	2	2	14
**Parthasarathy, 2018**	2	0	0	2	2	0	0	0	2	2	0	0	10
**Song, 2011**	2	0	0	2	2	0	0	0	2	2	0	2	12
**Vance, 2014**	2	NA	0	2	2	0	0	NA	NA	2	NA	0	8
**Wang, 2010**	2	2	0	2	0	NA	NA	0	2	2	0	2	12
**Wang, 2020**	2	2	0	2	2	0	0	0	2	2	0	0	12
**Yun, 2009**	2	0	0	2	0	NA	NA	0	2	2	0	2	10

NA: Not assessed.

## Data Availability

The data presented in this study are available on request from the corresponding author.

## Abbreviations

AMPK	AMP-activated protein kinase
COMP	Cartilage oligomeric matrix protein
CRP	C-reactive protein
DdRFP	matriptase sensitive protein biosensor based on dimerization-dependent red fluorescent protein
ECM	Articular cartilage extracellular matrix
ELISA	Enzyme-Linked Immunosorbent Assays
FBG	New fiber Bragg grating
FCD	fluid control device
FMGC	Fluoro-microbead guiding chip
FO-PPR	Fiber optic-particle plasmon resonance biosensor
GPI	antibodies against glucose 6-phosphate isomerase
IDE	Immunoassay with the specific antibody for uCTX-II
MIP	Molecular Imprinted Polymer sensor
MMP-1	Matrix metalloproteinase 1
MMP-3	Matrix metalloproteinase 3
OA	osteoarthritis
QCM	Quartz crystal microbalance
SAM	Biosensor based on label-free immuno-sensing with self-assembled monolayer
sCTx-I	Serum C-terminal telopeptide of type I collagen
sCTX-II	Serum C-terminal telopeptide fragment of type II collagen
SPRi	Plasmon resonance biosensor
TNF-a	Tumor necrosis factor.
uCTX-II	Urinary C-terminal telopeptide fragment of type II collagen
